# Formulation of Ebastine Fast-Disintegrating Tablet Using Coprocessed Superdisintegrants and Evaluation of Quality Control Parameters

**DOI:** 10.1155/2022/9618344

**Published:** 2022-05-19

**Authors:** Bhawana Dhakal, Jaybir Kumar Thakur, Reema Kumari Mahato, Ishwori Rawat, D. C. Rabin, Rahul Rana Chhetri, Kedar Prasad Shah, Atul Adhikari, Jitendra Pandey

**Affiliations:** ^1^Department of Pharmacy, Kantipur Academy of Health Sciences, Kathmandu 44600, Nepal; ^2^Department of Quality Control, Times Pharmaceuticals Private Limited, Chitwan 44200, Nepal; ^3^Department of Research and Development, Asian Pharmaceuticals Private Limited, Rupandehi 32900, Nepal; ^4^Department of Research and Development, Deurali Janata Pharmaceutical Private Limited, Kathmandu 44600, Nepal; ^5^Department of Pharmacy, Crimson College of Technology, Affiliated with Pokhara University, Devinagar-11, Butwal 32900, Nepal

## Abstract

Ebastine is a long-acting, nonsedating, second-generation antihistaminic drug that prevents histamine action, mainly in immediate hypersensitivity. This project was aimed to formulate and characterize orodispersible tablets of ebastine, utilizing different proportions of three disintegrants, namely crospovidone, sodium starch glycolate, and coprocessed superdisintegrant. Initially, fifteen trial batches of ebastine orodispersible tablets were outlined using the central composite design of Minitab software. The tablets were formulated by the direct compression method. The compressed tablets were then evaluated for precompression and postcompression physicochemical parameters, such as angle of repose, Carr's index, Hausner's ratio, hardness, thickness, weight variation, drug content, friability, wetting time, disintegration time, dispersion time, and water absorption ratio. The *in vitro* dissolution test was conducted according to Indian Pharmacopeia 2018, with the help of the rotating paddle method using 0.5% w/v sodium lauryl sulfate buffer in 0.1 N HCl. For the optimized batch (8^th^ batch), all the physicochemical parameters like angle of repose (33.77°), Carr's index (19.34%), Hausner's ratio (1.24), weight variation (202.5 mg), hardness (4.3 kg/cm^2^), friability (0.44%), thickness (3.16 mm), dissolution (95.78%), and drug content (101.67%) were within the acceptable limit as per Indian Pharmacopeia 2018. The wetting time, disintegration time, dispersion time, and water absorption ratio were reported to be 25.1 seconds, 16.0 seconds, 38.6 seconds, and 91.92%, respectively. Hence, the results suggested that orodispersible tablets of ebastine can be formulated. Furthermore, the mixing of crospovidone, sodium starch glycolate, and coprocessed super disintegrants can result in excellent desirable properties in the orodispersible tablet.

## 1. Introduction 

The oral drug delivery system is considered the most suitable, safest, and inexpensive method for drug administration. It is a convenient route for systemic effects as it enables easy ingestion, self-medication, accurate dosage, and patient compliance [1]. A major disadvantage of solid dosage forms is the difficulty in swallowing (dysphagia) or chewing in some patients (especially for geriatric and pediatric patients), gastrointestinal enzymatic degradation, and slow onset of action [2].

To overcome this problem, the formulations of tablets that can rapidly dissolve or disintegrate in the oral cavity are the best alternative. Fast dissolving tablets are also known as melt-in-mouth tablets, mouth-dissolving tablets, orodispersible tablets, quick-dissolving, porous tablets, etc. [3] The basic technique employed for the development of fast-dissolving tablets (FDT) is the use of superdisintegrants like crospovidone (polyplasdone), cross-linked carboxymethyl cellulose (croscarmellose), etc. They facilitate instantaneous tablet disintegration after placing on the tongue, ensuring drug release in saliva. Drugs absorbed through the “oral cavity” directly enter into systemic circulation via the jugular vein, leading to the instant onset of action, avoidance of presystemic metabolism, drug decomposition in the gastric region, and enzymatic hydrolysis in the intestine [4]. The main criteria for fast-dissolving tablets are to disintegrate or dissolve rapidly in the saliva present in the oral cavity within 15 to 60 seconds without the need for water and should have a pleasant mouthfeel [5].

Natural and synthetic superdisintegrants, such as mucilage cross-linked carboxymethyl cellulose (croscarmellose) and sodium starch glycolate, crospovidone, etc., provide the immediate disintegration of tablets and facilitate the design of the delivery system with desirable characteristics. These types of formulations are widely recommended for the drugs used in an emergency. e.g., cardiac agents, asthma, brain stroke, hyperlipidemia, etc. [6] During the formulation of any kind of tablet, one of the major challenges is to maintain desirable flow properties of powder mixture as we have to mix many excipients, which have diverse flowing properties. Sometimes, we have to add more amounts of some excipients (those excipients that can improve flow properties) than expected. While formulating tablets, if we use such type of single excipients that have multiple desirable properties, such as superior compressibility [2], better flow property [5], rapid disintegration capacity [3], taste-masking effect [4], and less moisture sensitivity [7], which are required for the quality formulation, then it will help to decrease the bulk of tablet, and it will have better pharmaceutical acceptability. Thus, nowadays, among different approaches, the preparation of coprocessed superdisintegrants is a popular technique [8]. In this technique, two or more superdisintegrants will interact at a subparticle level to form a new entity with diverse properties, such as taste masking effect, good flowability, desirable compressibility, excellent disintegration, and dissolution properties [6]. The coprocessed excipients are prepared using several techniques, such as freeze-drying, spray drying, cocrystallization, and wet granulation [5, 8]. Thus, newly formed coprocessed excipients result in the development of excipients granules with superior characters as compared to normal physical mixtures of excipients or individual components [9]. There are several reasons to prepare coprocessed excipients. Sometimes, it is necessary to prepare a powder mixture with a high degree of compressibility while formulating the tablets by the direct compression method. Besides, we have to mask the bitter test of tablets, especially for pediatric and geriatric patients to bring about acceptable palatability. Tablet formulation often requires the incorporation of a large range of functional excipients, such as fillers, sweeteners, dispersing agents, lubricants, etc. In this context, coprocessed excipients can be useful to reduce the number of separate excipients required within the formulation, thus diminishing extensive experiments. Furthermore, the preparation of coprocessed excipients is essential to improve flow properties, chemical stability, fill weight uniformity, dilution potential of the powder mixture, and reduce lubricant sensitivity [10, 11].

The formulation of the fast-dissolving tablets using coprocessed superdisintegrants will increase the water uptake with the shortest wetting time, and thus, it reduces the disintegration time [6, 12]. Coprocessing techniques minimize the drug particle adherence to the excipients and decrease the segregation. Some examples of coprocessed superdisintegrants are coprocessed microcrystalline cellulose and starch, croscarmellose sodium, crospovidone, microcrystalline cellulose, calcium phosphate dehydrate spray-dried lactose, maize starch, dibasic calcium phosphate dehydrates calcium carbonate, acacia, etc. [13]

Chemically, ebastine is characterized as 1-[4-(1, 1-Dimethylethyl) phenyl]-4-[4-(diphenyl methoxy)-1-piperidinyl]-1-butanone with an empirical formula C_32_H_39_NO_2_. It is available as a white powder, soluble in dichloromethane, slightly soluble in methanol, and insoluble in water. Its melting point is 86°C. It is highly permeable in the lipid membrane and classified as a biopharmaceutics classification system (BCS class II) [14]. Ebastine belongs to the class of drugs called nonsedative selective inhibitors of the histamine H1 receptor. Because of its inverse antagonizing effect, it prevents the action of histamine, majorly immediate hypersensitivity effects. It acts on the blood capillaries, bronchi, and some other smooth muscles. Thus, it is a very successful drug to prevent or alleviate motion sickness, seasonal rhinitis, and allergic dermatitis [14].

The use of ebastine has been increasing nowdays because of its nonsedating effect and selective inhibition of the histamine H1 receptor [14]. However, the conventional oral tablets of ebastine may have some problems related to its taste, difficulty in swallowing (dysphagia), chewing, the onset of action, the convenience of use, the novelty in the formulation, ease to take, ease to carry, etc. To overcome these problems, there are new drug delivery dosage forms known as oral disintegrating tablets (ODTs) [15]. These solid dosage forms can be dissolved or suspended with saliva in the mouth for easy swallowing. Generally, they disintegrate within 60 s or less, and the drug is absorbed through the local oral mucosal tissues or the gastrointestinal (GI) tract [5]. Among the different techniques of taste masking, the coprocessed super disintegrants approach has received considerable attention for pharmaceutical applications [12]. This technique enables the interaction of drug excipients at the subparticle level and provides a synergy of functionality improvement along with masking the unwanted properties of the individual (such as bitter taste) [6]. The formulation of the fast-dissolving tablets using coprocessed superdisintegrants will increase the water uptake with the shortest wetting time, and thus, it reduces the disintegration time [9, 13]. The objective of this study is to formulate the fast-disintegrating tablet of ebastine using the coprocessed superdisintegrants technique to mask the bitter taste of the tablet and achieve prompt dissolving of the tablet in a small amount of water or even in the unavailability of water so that newly formulated tablets can ensure the rapid dissolution of the drug and absorption, which may fascinate the rapid onset of action. This newly formulated fast-disintegrating tablet will be most suitable for elderly patients, paralyzed patients, infant patients, or bed-ridden patients who have swallowing problems. Moreover, in contrast to other studies of coprocessed superdisintegrating tablet formulations, our research work is focused on the formulation of ODTS by mixing coprocessed superdisintegrants and their physical mixtures and evaluating their quality control parameters.

## 2. Materials and Methods

### 2.1. Drugs and Chemical

Ebastine was obtained as a gift from Time Pharmaceuticals Pvt. Ltd, Nepal. The Maize starch and sodium starch glycolate were purchased from Himedia Laboratories India. Crospovidone and talc were purchased from Loba Chemie Pvt. Ltd, Mumbai. Magnesium stearate, aspartame, microcrystalline cellulose 112 (MCC 112), and talc were purchased from Sigma-Aldrich, Inc. (St Louis, MO, USA). Other chemical reagents were available at the Department of Pharmacy Kantipur Academy of Health Sciences. All the chemicals and reagents used were of analytical grade.

### 2.2. Instruments

UV spectrophotometer model UV-1601/SN-A10753984157 (Shimazu Corporation, Kyoto, Japan), USP dissolution apparatus, Electrolab, Model TDT-08L/SN-0205045, refrigerator (LG company), eectronic balance (FA1104 Electronic Balance), rectangular water bath (VIT company), FTIR, Agilent technology, Model Cary 630, Microprocessor pH meter, Hanna, Model pH 211, and tablet compression machine (punch) 1 station (Shiva Pharma Engineering India) were used in this study.

### 2.3. Drug-Excipients Compatibility Study

To ensure drug excipient compatibility, the infrared (IR) spectroscopy technique was employed using an FTIR spectrophotometer, and the spectrum was measured in the wavelength region of 1950 to 400 cm^−1^. The procedure consisted of spreading a sample (drug alone or the mixture of drug and all excipients) in potassium bromide and compressed into discs by applying a pressure of 5 tons for 5 min in a hydraulic press. The pellet was kept in the light path, and the spectrum was achieved [2].

### 2.4. Preparation of Coprocessed Superdisintegrant

The coprocessed superdisintegrant was prepared by solvent evaporation technique. At first, the mixture of crospovidone and sodium starch glycolate (in the ratio of 3 : 1) was blended properly and added to 65 mL of isopropyl alcohol. The contents of the beaker (250 mL capacity) were stirred with the help of a magnetic stirrer, maintaining the temperature between 65°C and 70°C, until almost all of the isopropyl alcohol was evaporated. Then, the wet coherent mass was subjected to granulation by passing through a 60-mesh sieve. After that, newly formed wet granules were dried in a tray dryer at 60°C for 20 minutes. Finally, the dried granules were sifted on a 60-mesh sieve and stored in an airtight container till further use [16].

### 2.5. Formulation of Fast-Disintegrating Tablets of Ebastine

The fast-disintegrating tablets of ebastine were prepared by the direct compression method. All the powders in pure form were accurately weighed. All ingredients were mixed step-by-step, passed through a sieve (number 60), and mixed with the drug for 15 min in a polybag. Lubricants, such as talc and magnesium stearate, were added to this powder mixture. Flavoring (mannitol) and a sweetening agent (aspartame) were added. At last, the final mixture was blended for 5 min. The active blends were then compressed into tablets with an average weight of 200 mg. The tablets were punched in a single station compression machine. The punch used for tablet compression was an 8.0 mm shallow round punch [16–18]. The details of the composition of each batch were calculated using the central composite design of Minitab software. While designing the batches, crospovidone, SSG, and coprocessed superdisintegrants were used as dependent variable ingredients. The upper and lower ranges of each excipient were optimized using the literature [19, 20]. As shown in Table 1, a total of 15 trial batches were designed.

### 2.6. Evaluation of Fast-Disintegrating Tablets

The quality control parameters of newly formulated tablets were evaluated using IP-2018 [21] and other literature [19, 22–24].

#### 2.6.1. Precompression Evaluation of Powder Blends

*(1) Bulk Density and Tapped Density*. The bulk and tapped density of precompression powder was calculated by equations (1) and (2) [23].

(1) $$\begin{array}{c} \text{Bulk density}=\frac{\text{Mass of powder}\left(\text{g}\right)}{\text{Bulk volume of powder in measuring cylinder}\left(\text{mL}\right)}, \end{array}$$

(2) $$\begin{array}{c} \text{Tapped density}=\frac{\text{Mass of powder}\left(\text{g}\right)}{\text{Tapped volume of powder in measuring cylinder}\left(\text{mL}\right)}. \end{array}$$

*(2) Angle of Repose*. The angle of repose gives the measurement of the maximum possible angle between the surface of the pile of powder and the horizontal plane. A simple funnel method was used to determine the angle of repose. For this, an accurately weighed powder blend was poured through a funnel that can be raised vertically. The funnel height was adjusted in such a way that the tip of the funnel just touched the apex of the powder heap. The powder was subjected to flow freely through the funnel onto the horizontal surface. After that, the diameter of the powder cone was determined and then its radius (*r*). The height of the pile (*h*) was also calculated accurately. Finally, the angle of repose was calculated using equation (3) [23]. The measurement was performed in triplicates, and the mean value was calculated. The relationship between flowability and angle of repose is given in Table 2 [25].


(3) $$\begin{array}{c} \text{Angle of repose}=\;\tan^{-1}\left(\text{h}/\text{r}\right). \end{array}$$

*(3) Carr's Index and Hausner's Ratio*. The flow characteristics of precompression powder were determined by measuring compressibility index/Carr's index and Hausner's ratio. Compressibility is the simplest way of measuring the flow property of powders. It is an indication of the ease with which materials can be induced to flow and is given by Carr's index (CI), which can be calculated from equation (4) [24]. The relationship between CI and flow character is given in Table 3 [26].

(4) $$\begin{array}{c} \text{Carr's index}=\frac{100\;\left(V_{0}-V_{f}\right)}{Vo}, \end{array}$$

where, *V*_0_ = unsettled apparent volume and *V*_*f*_ = final tapped volume.

Similarly, Hausner's ratio is an index of the flow properties of powders related to the interparticle friction and is calculated as shown in equation (5) [27]. The relationship between Hausner's ratio and flow character is given in Table 4 [28]. For the evaluation of precompression parameters, all the results were calculated in triplicate, and the mean value and SD were calculated. The results of precompression parameters evaluation are depicted in Table 5.

(5) $$\begin{array}{c} \text{Hausner's ratio}=\frac{V_{0}}{V_{f}}. \end{array}$$

#### 2.6.2. Postcompression Evaluation of the Tablet

Various postcompression parameters, namely hardness, thickness, friability, drug content uniformity, disintegration time, dispersion time, wetting time, water absorption ratio, and *in vitro* drug release study were evaluated by adopting the method described in IP 2018 [21].

*(1) Weight Variation Test*. Randomly, 20 tablets from each formulation were selected and weighed individually. The individual weights were compared with the mean weight, and standard deviations (SD) were calculated. The weight variation limits are depicted in Table 6 (IP 2018). To comply with Indian Pharmacopeia, not more than 2 of the individual tablets should deviate from average weight by more than the percentage described in Table 6 [21].

*(2) Thickness Variation*. Arbitrarily, five tablets from each formulation were taken, and their thicknesses were measured using the Vernier caliper. Then, the mean thickness and SD were calculated [21].

*(3) Tablet Hardness*. The resistance of tablets to shipping or breakage under the conditions of storage, transportation, and handling before usage depends on its hardness. The hardness of each batch of tablets was checked using a Monsanto hardness tester. The hardness was measured in terms of kg/cm^2^. For each batch, 5 tablets were selected randomly and tested for hardness. The average hardness of 5 tablets was measured, and SD was calculated [21].

*(4) Friability*. Friability generally refers to the loss in weight of tablets in the containers because of the removal of fines from the tablet surface. Friability generally reflects the poor cohesion of tablet ingredients. For this, the initial weights of these 20 tablets were recorded, placed in Roche friability, and rotated at the speed of 25 rpm for 100 revolutions. Then, tablets were removed from the friabilator, dusted off the fines, and again weighed. Finally, the percentage friability was calculated using equation (6) [21].

(6) $$\begin{array}{c} \text{\% Friability}=\frac{\text{Initial weight of tablets}-\text{Final weight of tablets }\left(\text{g}\right)}{\text{Initial weight of tablets}\left(\text{g}\right)}\;\times 100. \end{array}$$

**Figure 1:**
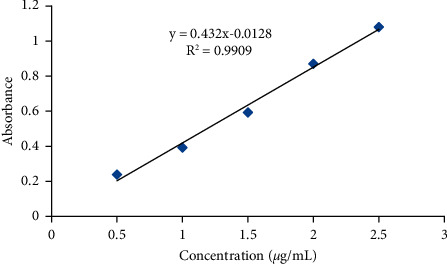
Standard calibration curve of ebastine.

*(5) Preparation of Ebastine Standard Solution for Calibration Curve*. The stock solution of standard ebastine was prepared in methanol at a concentration of 20 *μ*g/mL. Then, serial dilutions of ebastine (2.5 *μ*g/mL, 2 *μ*g/mL, 1.5 *μ*g/mL, 1 *μ*g/mL, and 0.5 *μ*g/mL) were prepared from the stock solution. Finally, these solutions were analyzed individually in triplicate using a UV spectrophotometer for the construction of the calibration curve. By plotting the mean absorbance (*y*-axis) versus concentration (*x*-axis), calibration equations were obtained [29]. The result of calibration curve plotting is given in Figure 1.

**Figure 2:**
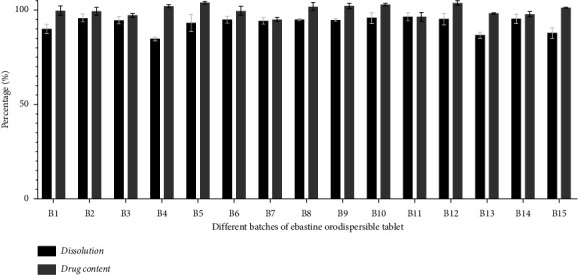
Bar diagram showing drug content and dissolution behavior of different batches.

*(6) Drug Content Evaluation*. For the assay of the newly formulated tablets, random 20 tablets were weighed and powdered. The powder, equivalent to 50 mg, was weighed accurately and dissolved in 100 mL of methanol. The solution was shaken thoroughly and sonicated for 15 minutes. The undissolved matters were removed by filtration through Whatman No.41 filter paper. The filtrate was diluted appropriately to prepare a final solution of 2 *µ*g/mL. The absorbance of the diluted solutions was measured at 254 nm using a UV spectrophotometer. The concentration of the drug was determined from the standard calibration curve of ebastine. For each batch, the assay was calculated in triplicate. Then, the mean assay and SD were calculated [21, 30, 31]. The results of the drug assay are depicted in Table 7 and Figure 2.

*(7) In Vitro Dissolution Studies*. The dissolution test was carried out according to the method described in IP 2018. In this method, the USP type II dissolution test apparatus was used at 37 ± 2°C and 50-rpm. A total of 900 mL of 0.5% w/v sodium lauryl sulfate buffer in 0.1 N HCl (pH-1.3) was used as dissolution medium. For each batch, six tablets were analyzed. An aliquot equal to 10 mL was withdrawn at 16 minutes [32]. The collected samples were filtrated. Then, 5 mL of filtrated solution was diluted to 50 mL using dissolution medium and subjected to analysis in the UV Spectrophotometer at 254 nm [21]. The cumulative % release of ebastine in the tablet sample was determined using a standard calibration curve of ebastine. Finally, the mean dissolution percentage and SD were calculated. The results of drug dissolution are depicted in Table 7 and Figure 2.

**Figure 3:**
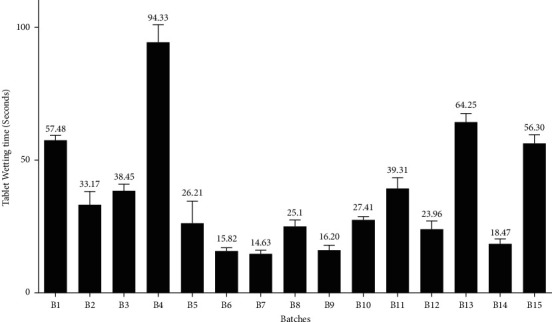
Bar diagram for the measurement of wetting time (in seconds) for different batches.

*(8) Wetting Time*. In the wetting time study, a piece of tissue paper that was folded twice was placed in a Petri dish (with an internal diameter of 9 cm), containing 9 mL of distilled water. A tablet was placed on the paper, and the time for the complete wetting of the tablet was measured in seconds. For each batch, the wetting time was determined in triplicate. Then, the mean wetting time and SD were calculated [17]. The results of the drug wetting time are depicted in Figure 3.

**Figure 4:**
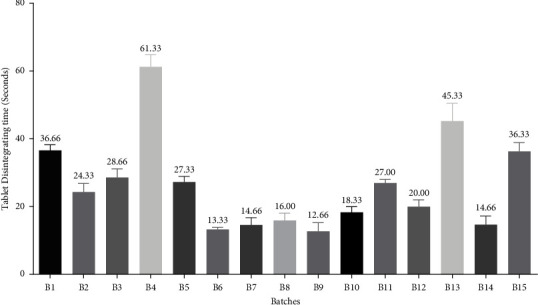
Bar diagram for the measurement of *in vitro* disintegration time (in seconds) for different batches.

*(9) In Vitro Disintegration Time*. In the disintegration time study, for each batch, three tablets were introduced in each tube of disintegration apparatus, and the tablet rack of the disintegration apparatus was positioned into a 1-liter beaker, containing 900 mL of distilled water. Then, the time of disintegration was recorded at 37 ± 2°C. Finally, the mean disintegration time and SD were calculated [33]. The results of *in vitro* disintegration time are depicted in Figure 4.

**Figure 5:**
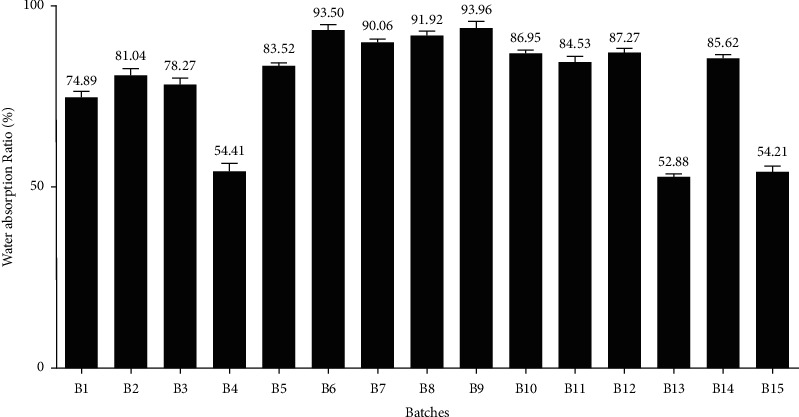
Bar diagram for the measurement of water absorption ratio (in percentage) for different batches.

*(10) Water Absorption Ratio*. A piece of tissue paper was folded twice and kept in a small Petri dish (with an internal diameter of 9 cm) containing 9 mL of water. For each Petri dish, each tablet was placed on the paper, and time consumed for complete wetting was noted. The completely wetted tablets were then weighed. Finally, the water absorption ratio (WR) was calculated using equation (7) [33]. Then, the mean water absorption ratio and SD were calculated [33]. The results of the water absorption ratio are depicted in Figure 5.

(7) $$\begin{array}{c} R=\frac{W_{a}-W_{b}\;}{W_{b}}\;\times 100, \end{array}$$

where *W*_*a*_ = weight of the tablet after absorption (mg), *W*_*b*_ = weight of the tablet before absorption (mg).

**Figure 6:**
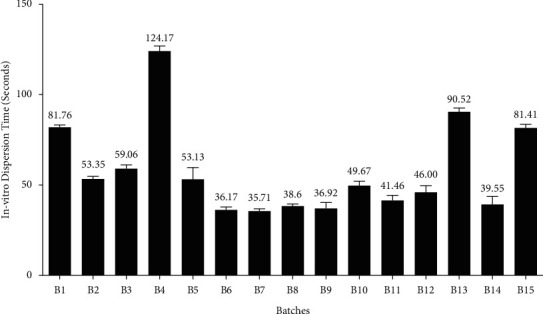
Bar diagram for the measurement of *in vitro* dispersion time (in seconds) for different batches.

*(11) In Vitro Dispersion Time*. *In vitro* dispersion time was measured by dropping a tablet in a Petri dish containing 10 mL of 6.8 pH phosphate buffer. The time taken for complete dispersion was noted. For each batch, dispersion time was determined in triplicate. Then, the mean dispersion time and SD were calculated [33]. The results of *in vitro* dispersion time are depicted in Figure 6.

### 2.7. Statistical Analysis

All the experiments were performed in triplicate, and the results were presented as mean ± SD. The statistical significance of differences for wetting time, dispersion time, disintegration time, and water absorption ratio were explored using a one-way analysis of variance (One-way ANOVA), with Tukey's post hoc test using GraphPad Prism 6.0 software. A *p*-value <0.05 was considered statistically significant.

## 3. Results and Discussion

### 3.1. Calibration Curve

For the calculation of drug content and dissolution profiles of different batches, the calibration curve equation (*Y* = 0.432*X* + 0.0128) was achieved by plotting absorbance versus concentration, ranging from 0.5 *µ*g/mL to 2.5 *µ*g/mL of a standard solution of ebastine in methanol (Figure 1). The absorbance was measured spectrophotometrically at 254 nm (in triplicates), and the correlation coefficient (*R*^2^) value was found to be 0.9909, which explains the positive correlation between the variables.

### 3.2. Drug Excipients Compatibility

**Figure 7:**
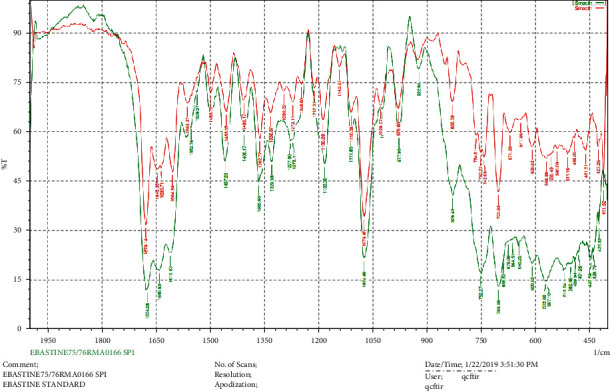
Chromatogram of FTIR for ebastine (green color: FTIR chromatogram of standard drug ebastine, red color: FTIR chromatogram of the ebastine mixed with all the excipients).

The analysis of the drug excipient compatibility studies was done by an FTIR spectrophotometer. The IR spectra of pure ebastine and the mixture of ebastine with all the excipients are shown in Figure 7. The major characteristic bands on the spectra of the pure compound and formulated tablets at 1674.28 cm^−1^, 1427.28 cm^−1^, 1365.66 cm^−1^, 1188.20 cm^−1^, 1070.40 cm^−1^, 977.95 cm^−1^, 829.43 cm^−1^, 752.27 cm^−1^, 703.08 cm^−1^, 572.88 cm^−1^, and 567.10 cm^−1^ were found to be similar. Besides, the absence of other peaks in the tablet spectra justified that there is no interaction [34].

### 3.3. Evaluation of Precompression Parameters

The evaluations of precompression parameters are given Table 5.

#### 3.3.1. Angle of Repose

Briefly, the angle of repose of different batches ranged from 27.73° to 36.7°. According to Table 2, all the trial batches revealed the passable flow property of the powder blend as given in Table 5.

#### 3.3.2. Bulk and Tapped Density

As shown in Table 5, the bulk density and tapped density ranged from 0.442–0.586 g/mL and 0.548–0.663 g/mL. Later, bulk density and tapped density were used to calculate Carr's index and Hausner's ratio.

#### 3.3.3. Carr's Index/Compressibility Index

Carr's index of all formulations ranged from 18.06% to 24.93%. According to Table 3, all the trial batches exhibited passable properties in terms of Carr's index. The data of all the batches are depicted in Table 5.

#### 3.3.4. Hausner's Ratio

It was determined by calculating the ratio of tapped to bulk density. Hausner's ratio of all formulations ranged from 1.22 to 1.35. While comparing the data with Table 4, only B13 did not show passable flow properties. The data of all the batches are depicted in Table 5.

Overall, the result from the preformulation study of 15 different batches suggested that only one batch, namely B13, was reported to be unsuitable for the formulation. The blend mixture that cannot pass the preformulation criteria may create trouble by sticking on the surface of hopper while doing the formulation of batches on a large scale [17].

### 3.4. Evaluation of Postcompression Parameters

#### 3.4.1. Weight Variation

From each batch, twenty tablets were randomly selected, and each was accurately weighed on the analytical balance. The average weight of the tablet was found to be between 198.07 and 203.79 mg. As shown in Table 7, the results of the weight variation were observed to be within the limit as indicated in the IP 2018 [21], as seen in Table 6.

#### 3.4.2. Friability

All orodispersible tablets of ebastine did not break or show any capping during the test. The friability of tablets was within the limit according to IP 2018 [21]. A maximum weight loss was not more than 1% of the weight of the tablet being tested. B4 was found to have the maximum friability (0.877%), and the minimum was observed in B10 (0.219%), as given in Table 7. It indicated that all the formulated tablets possess sufficient mechanical strength.

#### 3.4.3. Tablet Hardness

Using the Monsanto hardness tester, the hardness of the tablets was tested, and the results are tabulated in Table 7. B1 and B2 were found to have a maximum and minimum hardness of 4.5 kg/cm^2^ and 3.2 kg/cm^2^, respectively. The hardness of the tablets of all formulations was found to be in the range of 3.2 to 4.5 kg/cm^2^, which falls within the limit according to the previous study [35]. Mechanical integrity is of foremost importance in the successful formulation. The hardness of ODT is normally acceptable between 2 kg/cm^2^ and 8 kg/cm^2^. The hardness of tablets varied according to the force applied during tablet compression along with the quantity and chemical nature of the binding agent utilized. During the formulation, a constant compression force was applied for all the batches. Therefore, the change in the hardness values of different ODTs observed in Table 7 could be because of the quantity and type of binding agents in the coprocessed excipients [17]. Our study revealed that the increased concentration of SSG can greatly increase the hardness of tablets (B1 and B5). Crospovidone also has a direct effect on increasing hardness, however, its effect is moderate as compared to SSG. It is to be noted that the coprocessed superdisintegrant played a crucial role to maintain the hardness of the tablet, because at very low concentration, it has no effect on hardness, and when its amount is increased to moderate, it can increase hardness. Surprisingly, when the amount of coprocessed superdisintegrant is very high, its hardness is reported to be reduced again (B2).

#### 3.4.4. Tablet Thickness

The thicknesses of all the formulations were in the range of 2.99 to 3.36 mm (Table 7). As the tablet thickness of each formulation is almost similar, it can be predicted that the powder blend was consistent because of the uniform particle size [36].

#### 3.4.5. Drug Content

An assay is an investigative procedure for qualitatively assessing or quantitatively measuring the presence, amount, or the functional activity of an analyte [36]. The percentage of drug content was determined spectrophotometrically by measuring the absorbance at 254 nm with the help of the ebastine standard calibration curve. The percentage drug content of the formulation was found to be between 94.8% and 103.71% (Table 7). The maximum percentage content was reported on B12. Furthermore, the drug content for all the investigated batches complied with the limit (90–110%) given by IP 2018 [21]. Moreover, the drug content of all the batches was almost similar.

#### 3.4.6. *In Vitro* Drug Dissolution

To investigate the effect of superdisintegrants composition and amount in drug release pattern, the *in vitro* dissolution of newly formulated batches of ebastine orodispersible tablets was conducted, and the results are depicted in Table 7. As shown in Figure 2, the dissolution percentage of different batches was in the range of 84.66% to 96.27%. Our data revealed that the dissolution pattern of most of the batches was almost similar. However, for some batches with higher dispersion, disintegration, and dispersion time, the dissolution percentages were reported to be comparatively low.

#### 3.4.7. Wetting Time

The results of the wetting time analysis for all the batches are depicted in Figure 3. All the batches gave an acceptable result for wetting time analysis (<180 sec) [17]. Among them, B4 and B9 were found to have a maximum and minimum wetting time of 94.33 sec and 14.63 sec, respectively. The wetting time is a very significant parameter for the disintegration behaviors of the ODTs. Wetting is directly related to the gross hydrophilicity of the excipients and the internal structure of tablets [17]. For ODTs, the measurement of the wetting time is necessary to understand the swelling tendency of superdisintegrants, even in the presence of a little amount of water [37]. In this study, all the batches contained a varied proportion of crospovidone, SSG, and coprocessed superdisintegrants. Therefore, the individual effect of these disintegrants on wetting time could not access properly. However, for most of the batches, long wetting time was reported where the proportion of SSG is comparatively high. Also, the wetting time was longer for the tablets with higher hardness. Increased hardness always indicates the extent of compactness for the tablets. Because of relatively higher compactness in the tablets with increased hardness, it may render the water penetration rate and may prolong the wetting time [38].

#### 3.4.8. *In Vitro* Disintegration Time

The results of *in vitro* disintegration time measurement for all batches are presented in Figure 4. Among them, B4 and B9 were found to have a maximum and minimum disintegration time of 61.33 sec and 12.66 sec, respectively. The spontaneous or partial disintegration of tablets is an indication of the low bioavailability of that drug when administered by patients. According to the previous studies, the orally disintegrating tablets should disintegrate completely in the mouth within 1 min or less, ideally about 30 s or less [39]. Therefore, in this study, only B4 (DT: 61.33 sec) was reported to be out of the specified limit of disintegration time.

It is to be noted that a direct correlation was found between wetting time and disintegration time among all the batches. Thus, disintegration time was reported to be increased with an increase in the wetting time and vice versa. The effect of water-soluble excipients and disintegrants is always a governing factor for the disintegration time of any tablet [17]. Crospovidone increases water uptake in the tablets by a swelling and wicking process, drawing water in the tablet by a capillary action associated with its porous morphology, resulting in the breaking of interparticle bonds and causing prompt disintegration [40]. Also, it was reported that SSG and crospovidone had prompt capillary activity and significant hydration power with negligible affinity to gel formation [41]. It is to be noted that both of the superdisintegrants used in our study are cross-linking agents. The water uptake by these disintegrants relies on their various chemical attributes, such as the extent of hydroxylation, cross-linking, and carboxymethylation. The cross-linking process renders their solubility in water and diminishes the viscosity of adjacent water, thus attaining greater drug release. Furthermore, the presence of hydroxyl group in these superdisintegrants results in the formation of a strong hydrogen-bonded network, which lessens water penetration into polymers. However, when these groups are partly replaced by carboxymethyl or similar types of hydrophobic groups, the generation of the hydrogen bond is deranged, permitting water entry into polymers. Besides, some of these superdisintegrants also possess salt impurities like sodium citrate and/or sodium chloride, which enable the prompt entry of water into the polymer, thus easing dissolution [42]. Moreover, the incorporation of two potent superdisintegrants along with coprocessed superdisintegrants in every batch might be the main reason for excellent DT in most of the batches.

#### 3.4.9. Water Absorption Ratio

As depicted in Figure 5, the water absorption ratio of different batches was reported to be in the range of 52.88% to 93.50%. For all the batches, the water absorption ratio exhibited an inverse relation with the wetting time and disintegration time. It is meant to say that water absorption was higher for the batch that has a lower value of wetting time and DT, and vice versa. The water absorption ratio also is an important parameter to understand the potency of disintegrants to swell even in little quantity of water, which later fascinates the dissolution of the drug [43].

#### 3.4.10. *In Vitro* Dispersion Time

The *in vitro* dispersion time of different batches was reported to be in the range of 36.17 sec to 124.17 (Figure 6). For all the batches, the *in vitro* dispersion time exhibited proportional relation with wetting time and disintegration time. It is meant to say that dispersion time was higher for the batches that have higher values of wetting time and DT, and vice versa.

In this way, the evaluation of preformulation and postformulation of fifteen different trial batches indicated that a total of 13 batches (except B4 and B13) passed all the criteria given by pharmacopeia. Among them, batch B8 was considered to be the optimized formulation as it gave excellent results in different evaluation parameters, such as hardness (4.3 Kg/cm^2^), friability (0.44%), assay (101.67%), dissolution (95.78%), wetting time (25.1 sec), *in vitro* disintegrating time (16.0 sec), *in vitro* dispersion time (38.6 sec), and water absorption ratio (91.92%). While comparing with the previous study, the dissolution profile of the optimized batch was found to be better than that of ebastine oral dispersible tablet (75% drug release) prepared by the molecular dispersion method [34]. Furthermore, in a previous study, ebastine tablets were prepared using the surface solid dispersion method, where croscarmellose sodium was incorporated as a hydrophilic water-insoluble carrier. The drug content and dissolution profile (in 60 min) of the optimized formulation in that study were reported to be 98.39% and 93.19%, respectively [44]. It signified that ODTs prepared in our study had a similar drug release profile and better drug content than ebastine tablets prepared by using the solid dispersion technique. However, the extensive study of optimized batches, such as the evaluation of pharmacokinetic parameters in human volunteers, real-time and accelerated stability study, the study of drug release kinetic model, and drug-excipient compatibility study using differential scanning calorimetry (DSC), is recommended for further studies.

## 4. Conclusion

This research project was conducted to formulate and evaluate orodispersible tablets of ebastine with rapid release properties to achieve patient compliance for the management of different types of allergic conditions. The evaluation of optimized batches revealed the acceptable precompression parameters along with a low value of DT (16.0 sec), dispersion time (38.6 sec), wetting time (25.1 sec), and sufficient water absorption capacity (91.9%). Furthermore, cumulative drug release within 16 min (95.78%) and drug content (101.67%) were excellent as per pharmacopeia limit, and it fulfilled the criteria of an ideal disintegrating tablet. Many studies have been conducted to prove the better effect of coprocessed superdisintegrants as compared to the physical mixture of superdisintegrants. The results of our study concluded that the formulation of ODTS by mixing coprocessed superdisintegrants and their physical mixture can produce excellent desirable properties.

## Figures and Tables

**Table 1:** Composition of various batches of fast disintegrating tablets of ebastine designed by Minitab software.

	EBS (mg)	MS (mg)	CPV (mg)	SSG (mg)	CPS (mg)	Talc (mg)	Mannitol (mg)	APM (mg)	MCC112 (mg)	MgS (mg)
B1	20	69.924	5.425	15.57	6.58	3	45	10	23	1.5
B2	20	71.892	5.425	7.75	12.432	3	45	10	23	1.5
B3	20	74.25	7.75	12.4	3.1	3	45	10	23	1.5
B4	20	85.565	5.425	0.07	6.58	3	45	10	23	1.5
B5	20	78.9	3.1	12.4	3.1	3	45	10	23	1.5
B6	20	67.9	7.75	12.4	10.06	3	45	10	23	1.5
B7	20	81.24	3.1	3.1	6.58	3	45	10	23	1.5
B8	20	77.745	5.425	7.75	6.58	3	45	10	23	1.5
B9	20	88.2	3.1	3.1	3.1	3	45	10	23	1.5
B10	20	81.655	1.514	7.75	6.58	3	45	10	23	1.5
B11	20	76.59	7.75	3.1	6.58	3	45	10	23	1.5
B12	20	83.55	7.75	3.1	10.06	3	45	10	23	1.5
B13	20	73.834	9.335	7.75	6.58	3	45	10	23	1.5
B14	20	83.597	5.425	7.75	0.727	3	45	10	23	1.5
B15	20	71.94	3.1	12.4	10.06	3	45	10	23	1.5

(EBS: ebastine, MS: maize starch, CPV: crospovidone, SSG: sodium starch glycolate, CPS: coprocessed superdisintegrant, MCC112: microcrystalline cellulose 112, Mgs: magnesium stearate, and APM: aspartame).

**Table 2:** Effect of angle of repose on flow character.

Angle of repose	Flow character
<25°	Excellent
25–30°	Good
30–40°	Passable
>40°	Very poor

**Table 3:** Effect of Carr's index on flow character.

Carr's index (%)	Flow character
<10	Excellent
11–15	Good
16–20	Fair
21–25	Passable
26–31	Poor
32–37	Very poor
>37	Very very poor

**Table 4:** Effect of Hausner's ratio on flow character.

Hausner's ratio	Flow character
1.00–1.11	Excellent
1.12–1.18	Good
1.19–1.25	Fair
1.26–1.34	Passable
1.35–1.45	Poor
1.46–1.59	Very poor
>1.600	Very-very poor

**Table 5:** Evaluation of precompression parameters of granules of fifteen different trial batches.

Formulations	Bulk density (g.mL^−1^)	Tapped density (g.mL^−1^)	Carr's index (%)	Hausner's ratio	Angle of repose (*θ*)
B1	0.53 ± 0.008	0.663 ± 0.014	20.13 ± 0.18	1.25 ± 0.003	33.43 ± 0.25
B2	0.496 ± 0.004	0.653 ± 0.005	24.07 ± 0.19	1.31 ± 0.003	36.70 ± 0.28
B3	0.487 ± 0.002	0.647 ± 0.001	24.70 ± 0.43	1.32 ± 0.007	35.90 ± 0.20
B4	0.491 ± 0.002	0.655 ± 0.005	24.93 ± 0.22	1.33 ± 0.004	33.06 ± 0.77
B5	0.494 ± 0.004	0.638 ± 0.006	22.46 ± 0.74	1.29 ± 0.012	31.53 ± 0.32
B6	0.483 ± 0.002	0.641 ± 0.008	24.64 ± 0.48	1.33 ± 0.008	34.26 ± 0.90
B7	0.481 ± 0.002	0.637 ± 0.005	24.42 ± 0.43	1.32 ± 0.007	31.43 ± 1.05
B8	0.442 ± 0.006	0.548 ± 0.002	19.34 ± 1.57	1.24 ± 0.024	33.77 ± 0.63
B9	0.443 ± 0.005	0.555 ± 0.003	20.27 ± 1.46	1.25 ± 0.022	32.97 ± 0.44
B10	0.46 ± 0.021	0.579 ± 0.002	20.63 ± 1.77	1.26 ± 0.03	33.54 ± 0.59
B11	0.486 ± 0.02	0.590 ± 0.002	18.06 ± 0.41	1.22 ± 0.002	27.9 ± 0.3
B12	0.477 ± 0.004	0.596 ± 0.006	19.98 ± 1.80	1.25 ± 0.028	27.73 ± 0.7
B13	0.478 ± 0.005	0.647 ± 0.005	26.11 ± 1.58	1.35 ± 0.029	33.96 ± 0.17
B14	0.586 ± 0.003	0.613 ± 0.001	20.58 ± 1.14	1.26 ± 0.018	32.71 ± 0.75
B15	0.494 ± 0.003	0.654 ± 0.002	24.45 ± 0.80	1.32 ± 0.014	31.83 ± 0.40

**Table 6:** Weight variation limit.

Average weight (mg)	Maximum difference (%)
84 or less	10
84–250	7.5
>250	5

**Table 7:** Evaluated postcompression parameters of fifteen different trial batches.

Batches	Weight variation (mg±SD)	Friability (%)	Hardness (kg/cm^2^ ± SD)	Thickness (mm ± SD)	Drug content (% ± SD)	Dissolution (%)
B1	200.05 ± 1.98	0.443	4.5 ± 0.57	3.04 ± 0.01	99.51 ± 2.49	89.83 ± 2.44
B2	199.71 ± 1.77	0.398	3.2 ± 0.27	3.01 ± 0.02	99.01 ± 2.10	95.61 ± 2.1
B3	199.81 ± 2.24	0.209	3.8 ± 0.44	3.02 ± 0.01	96.92 ± 0.93	94.34 ± 1.97
B4	198.07 ± 2.12	0.877	3.6 ± 0.41	2.99 ± 0.08	101.82 ± 0.83	84.66 ± 0.95
B5	200.09 ± 2.01	0.318	4.4 ± 0.41	3.36 ± 0.03	103.71 ± 0.59	94.55 ± 1.66
B6	200.85 ± 2.05	0.617	3.9 ± 0.65	3.15 ± 0.07	99.24 ± 2.45	94.67 ± 1.82
B7	202.06 ± 2.68	0.274	4.2 ± 0.27	3.04 ± 0.04	94.80 ± 0.93	94.09 ± 1.7
B8	202.5 ± 1.40	0.443	4.3 ± 0.27	3.16 ± 0.18	101.67 ± 2.10	95.78 ± 0.61
B9	202.46 ± 4.31	0.295	3.7 ± 0.75	3.25 ± 0.27	101.86 ± 1.48	94.63 ± 0.62
B10	202.77 ± 2.15	0.219	3.8 ± 0.57	3.27 ± 0.38	102.63 ± 0.72	95.65 ± 2.87
B11	201.47 ± 2.82	0.362	3.9 ± 0.41	3.15 ± 0.18	96.23 ± 2.27	96.27 ± 2.10
B12	202.14 ± 2.53	0.361	3.66 ± 0.11	3.15 ± 0.11	103.48 ± 1.16	94.98 ± 3.00
B13	198.36 ± 2.63	0.310	4.42 ± .23	3.15 ± 0.23	98.00 ± 0.04	86.58 ± 1.56
B14	203.79 ± 2.32	0.368	3.68 ± 0.13	3.34 ± 0.19	97.54 ± 1.33	95.17 ± 2.44
B15	202.52 ± 2.44	0.466	3.8 ± 0.15	3.15 ± 0.17	101.01 ± 0.20	87.70 ± 2.79

## Data Availability

All the data used to support the result of this research are available from J. Pandey upon request.

## Supplementary Materials

Supplementary Materials The data of statistical analysis applied for the comparison of different trial batches are presented in Supplementary Table S1.
