# Supplementary material for: Formulation of Ebastine Fast-Disintegrating Tablet Using Coprocessed Superdisintegrants and Evaluation of Quality Control Parameters
Source: ScientificWorldJournal. 2022 May 19;2022:9618344. doi: 10.1155/2022/9618344 (PMC9135552; doi:10.1155/2022/9618344)
Supplement: Supplementary Materials — The data of statistical analysis applied for the comparison of different trial batches are presented in Supplementary Table S1. [file 9618344.f1.docx]

TABLE S1: Statistical analysis of wetting time of different batches conducted by a one-way analysis of variance (One-way ANOVA), with Tukey post hoc test

| One-way analysis of variance | | | | | |
| --- | --- | --- | --- | --- | --- |
| P value | | < 0.0001 | |  |  |
| P value summary | | **** | |  |  |
| Are means signif. Different?  (P < 0.05) | | Yes | |  |  |
| Number of groups | | 15 | |  |  |
| F | | 108.4 | |  |  |
| R square | | 0.9806 | |  |  |
|  | |  | |  |  |
| ANOVA Table | | SS | | df | MS |
| Treatment (between columns) | | 21262 | | 14 | 1519 |
| Residual (within columns) | | 420.4 | | 30 | 14.01 |
| Total | | 21683 | | 44 |  |
| Tukey's Multiple Comparison Test | |  | |  |  |
| Tests | Mean Diff. | q | Significant?  (P < 0.05?) | Summary | 95% CI of diff |
| B1 vs B2 | 24.31 | 11.25 | Yes | *** | 13.04 to 35.57 |
| B1 vs B3 | 19.03 | 8.807 | Yes | *** | 7.771 to 30.30 |
| B1 vs B4 | -36.85 | 17.05 | Yes | *** | -48.11 to -25.59 |
| B1 vs B5 | 31.27 | 14.47 | Yes | *** | 20.00 to 42.53 |
| B1 vs B6 | 41.66 | 19.28 | Yes | *** | 30.40 to 52.93 |
| B1 vs B7 | 42.85 | 19.83 | Yes | *** | 31.59 to 54.11 |
| B1 vs B8 | 32.38 | 14.98 | Yes | *** | 21.12 to 43.65 |
| B1 vs B9 | 41.28 | 19.1 | Yes | *** | 30.01 to 52.54 |
| B1 vs B10 | 30.07 | 13.91 | Yes | *** | 18.81 to 41.33 |
| B1 vs B11 | 18.17 | 8.409 | Yes | *** | 6.911 to 29.44 |
| B1 vs B12 | 33.52 | 15.51 | Yes | *** | 22.26 to 44.79 |
| B1 vs B13 | -6.77 | 3.132 | No | ns | -18.03 to 4.492 |
| B1 vs B14 | 39.01 | 18.05 | Yes | *** | 27.75 to 50.27 |
| B1 vs B15 | 1.18 | 0.546 | No | ns | -10.08 to 12.44 |
| B2 vs B3 | -5.273 | 2.44 | No | ns | -16.54 to 5.989 |
| B2 vs B4 | -61.16 | 28.3 | Yes | *** | -72.42 to -49.89 |
| B2 vs B5 | 6.96 | 3.22 | No | ns | -4.302 to 18.22 |
| B2 vs B6 | 17.36 | 8.031 | Yes | *** | 6.095 to 28.62 |
| B2 vs B7 | 18.54 | 8.58 | Yes | *** | 7.281 to 29.81 |
| B2 vs B8 | 8.077 | 3.737 | No | ns | -3.185 to 19.34 |
| B2 vs B9 | 16.97 | 7.852 | Yes | *** | 5.708 to 28.23 |
| B2 vs B10 | 5.763 | 2.667 | No | ns | -5.499 to 17.03 |
| B2 vs B11 | -6.133 | 2.838 | No | ns | -17.40 to 5.129 |
| B2 vs B12 | 9.217 | 4.265 | No | ns | -2.045 to 20.48 |
| B2 vs B13 | -31.08 | 14.38 | Yes | *** | -42.34 to -19.81 |
| B2 vs B14 | 14.7 | 6.803 | Yes | ** | 3.441 to 25.97 |
| B2 vs B15 | -23.13 | 10.7 | Yes | *** | -34.39 to -11.86 |
| B3 vs B4 | -55.88 | 25.86 | Yes | *** | -67.15 to -44.62 |
| B3 vs B5 | 12.23 | 5.66 | Yes | * | 0.9712 to 23.50 |
| B3 vs B6 | 22.63 | 10.47 | Yes | *** | 11.37 to 33.89 |
| B3 vs B7 | 23.82 | 11.02 | Yes | *** | 12.55 to 35.08 |
| B3 vs B8 | 13.35 | 6.177 | Yes | ** | 2.088 to 24.61 |
| B3 vs B9 | 22.24 | 10.29 | Yes | *** | 10.98 to 33.51 |
| B3 vs B10 | 11.04 | 5.107 | No | ns | -0.2255 to 22.30 |
| B3 vs B11 | -0.86 | 0.3979 | No | ns | -12.12 to 10.40 |
| B3 vs B12 | 14.49 | 6.705 | Yes | ** | 3.228 to 25.75 |
| B3 vs B13 | -25.8 | 11.94 | Yes | *** | -37.07 to -14.54 |
| B3 vs B14 | 19.98 | 9.243 | Yes | *** | 8.715 to 31.24 |
| B3 vs B15 | -17.85 | 8.261 | Yes | *** | -29.12 to -6.591 |
| B4 vs B5 | 68.12 | 31.52 | Yes | *** | 56.85 to 79.38 |
| B4 vs B6 | 78.51 | 36.33 | Yes | *** | 67.25 to 89.78 |
| B4 vs B7 | 79.7 | 36.88 | Yes | *** | 68.44 to 90.96 |
| B4 vs B8 | 69.23 | 32.03 | Yes | *** | 57.97 to 80.50 |
| B4 vs B9 | 78.13 | 36.15 | Yes | *** | 66.86 to 89.39 |
| B4 vs B10 | 66.92 | 30.96 | Yes | *** | 55.66 to 78.18 |
| B4 vs B11 | 55.02 | 25.46 | Yes | *** | 43.76 to 66.29 |
| B4 vs B12 | 70.37 | 32.56 | Yes | *** | 59.11 to 81.64 |
| B4 vs B13 | 30.08 | 13.92 | Yes | *** | 18.82 to 41.34 |
| B4 vs B14 | 75.86 | 35.1 | Yes | *** | 64.60 to 87.12 |
| B4 vs B15 | 38.03 | 17.6 | Yes | *** | 26.77 to 49.29 |
| B5 vs B6 | 10.4 | 4.811 | No | ns | -0.8655 to 21.66 |
| B5 vs B7 | 11.58 | 5.36 | Yes | * | 0.3212 to 22.85 |
| B5 vs B8 | 1.117 | 0.5167 | No | ns | -10.15 to 12.38 |
| B5 vs B9 | 10.01 | 4.632 | No | ns | -1.252 to 21.27 |
| B5 vs B10 | -1.197 | 0.5537 | No | ns | -12.46 to 10.07 |
| B5 vs B11 | -13.09 | 6.058 | Yes | * | -24.36 to -1.831 |
| B5 vs B12 | 2.257 | 1.044 | No | ns | -9.005 to 13.52 |
| B5 vs B13 | -38.04 | 17.6 | Yes | *** | -49.30 to -26.77 |
| B5 vs B14 | 7.743 | 3.583 | No | ns | -3.519 to 19.01 |
| B5 vs B15 | -30.09 | 13.92 | Yes | *** | -41.35 to -18.82 |
| B6 vs B7 | 1.187 | 0.5491 | No | ns | -10.08 to 12.45 |
| B6 vs B8 | -9.28 | 4.294 | No | ns | -20.54 to 1.982 |
| B6 vs B9 | -0.3867 | 0.1789 | No | ns | -11.65 to 10.88 |
| B6 vs B10 | -11.59 | 5.364 | Yes | * | -22.86 to -0.3312 |
| B6 vs B11 | -23.49 | 10.87 | Yes | *** | -34.75 to -12.23 |
| B6 vs B12 | -8.14 | 3.766 | No | ns | -19.40 to 3.122 |
| B6 vs B13 | -48.43 | 22.41 | Yes | *** | -59.70 to -37.17 |
| B6 vs B14 | -2.653 | 1.228 | No | ns | -13.92 to 8.609 |
| B6 vs B15 | -40.48 | 18.73 | Yes | *** | -51.75 to -29.22 |
| B7 vs B8 | -10.47 | 4.843 | No | ns | -21.73 to 0.7955 |
| B7 vs B9 | -1.573 | 0.728 | No | ns | -12.84 to 9.689 |
| B7 vs B10 | -12.78 | 5.913 | Yes | * | -24.04 to -1.518 |
| B7 vs B11 | -24.68 | 11.42 | Yes | *** | -35.94 to -13.41 |
| B7 vs B12 | -9.327 | 4.315 | No | ns | -20.59 to 1.935 |
| B7 vs B13 | -49.62 | 22.96 | Yes | *** | -60.88 to -38.36 |
| B7 vs B14 | -3.84 | 1.777 | No | ns | -15.10 to 7.422 |
| B7 vs B15 | -41.67 | 19.28 | Yes | *** | -52.93 to -30.41 |
| B8 vs B9 | 8.893 | 4.115 | No | ns | -2.369 to 20.16 |
| B8 vs B10 | -2.313 | 1.07 | No | ns | -13.58 to 8.949 |
| B8 vs B11 | -14.21 | 6.575 | Yes | ** | -25.47 to -2.948 |
| B8 vs B12 | 1.14 | 0.5275 | No | ns | -10.12 to 12.40 |
| B8 vs B13 | -39.15 | 18.12 | Yes | *** | -50.42 to -27.89 |
| B8 vs B14 | 6.627 | 3.066 | No | ns | -4.635 to 17.89 |
| B8 vs B15 | -31.2 | 14.44 | Yes | *** | -42.47 to -19.94 |
| B9 vs B10 | -11.21 | 5.185 | No | ns | -22.47 to 0.05550 |
| B9 vs B11 | -23.1 | 10.69 | Yes | *** | -34.37 to -11.84 |
| B9 vs B12 | -7.753 | 3.587 | No | ns | -19.02 to 3.509 |
| B9 vs B13 | -48.05 | 22.23 | Yes | *** | -59.31 to -36.78 |
| B9 vs B14 | -2.267 | 1.049 | No | ns | -13.53 to 8.995 |
| B9 vs B15 | -40.1 | 18.55 | Yes | *** | -51.36 to -28.83 |
| B10 vs B11 | -11.9 | 5.505 | Yes | * | -23.16 to -0.6345 |
| B10 vs B12 | 3.453 | 1.598 | No | ns | -7.809 to 14.72 |
| B10 vs B13 | -36.84 | 17.05 | Yes | *** | -48.10 to -25.58 |
| B10 vs B14 | 8.94 | 4.137 | No | ns | -2.322 to 20.20 |
| B10 vs B15 | -28.89 | 13.37 | Yes | *** | -40.15 to -17.63 |
| B11 vs B12 | 15.35 | 7.102 | Yes | ** | 4.088 to 26.61 |
| B11 vs B13 | -24.94 | 11.54 | Yes | *** | -36.21 to -13.68 |
| B11 vs B14 | 20.84 | 9.641 | Yes | *** | 9.575 to 32.10 |
| B11 vs B15 | -16.99 | 7.863 | Yes | *** | -28.26 to -5.731 |
| B12 vs B13 | -40.29 | 18.64 | Yes | *** | -51.56 to -29.03 |
| B12 vs B14 | 5.487 | 2.539 | No | ns | -5.775 to 16.75 |
| B12 vs B15 | -32.34 | 14.97 | Yes | *** | -43.61 to -21.08 |
| B13 vs B14 | 45.78 | 21.18 | Yes | *** | 34.52 to 57.04 |
| B13 vs B15 | 7.95 | 3.678 | No | ns | -3.312 to 19.21 |
| B14 vs B15 | -37.83 | 17.5 | Yes | *** | -49.09 to -26.57 |

TABLE S2: Statistical analysis of dispersion time of different batches conducted by a one-way analysis of variance (One-way ANOVA), with Tukey post hoc test.

| **One-way analysis of variance** | | | | | |
| --- | --- | --- | --- | --- | --- |
| P value | | < 0.0001 | |  |  |
| P value summary | | **** | |  |  |
| Are means signif. Different?  (P < 0.05) | | Yes | |  |  |
| Number of groups | | 15 | |  |  |
| F | | 235.1 | |  |  |
| R square | | 0.991 | |  |  |
|  | |  | |  |  |
| ANOVA Table | | SS | | df | MS |
| Treatment (between columns) | | 27648 | | 14 | 1975 |
| Residual (within columns) | | 252 | | 30 | 8.4 |
| Total | | 27900 | | 44 |  |
| **Tukey's Multiple Comparison Test** | |  | |  |  |
| Tests | Mean Diff. | q | Significant?  (P < 0.05?) | Summary | 95% CI of diff |
| B1 vs B2 | 28.41 | 16.98 | Yes | *** | 19.69 to 37.13 |
| B1 vs B3 | 22.71 | 13.57 | Yes | *** | 13.99 to 31.43 |
| B1 vs B4 | -42.4 | 25.34 | Yes | *** | -51.12 to -33.68 |
| B1 vs B5 | 28.63 | 17.11 | Yes | *** | 19.91 to 37.35 |
| B1 vs B6 | 45.59 | 27.24 | Yes | *** | 36.87 to 54.31 |
| B1 vs B7 | 46.05 | 27.52 | Yes | *** | 37.33 to 54.77 |
| B1 vs B8 | 43.17 | 25.8 | Yes | *** | 34.45 to 51.89 |
| B1 vs B9 | 44.84 | 26.8 | Yes | *** | 36.12 to 53.56 |
| B1 vs B10 | 32.1 | 19.18 | Yes | *** | 23.38 to 40.82 |
| B1 vs B11 | 40.31 | 24.09 | Yes | *** | 31.59 to 49.03 |
| B1 vs B12 | 35.76 | 21.37 | Yes | *** | 27.04 to 44.48 |
| B1 vs B13 | -8.757 | 5.233 | Yes | * | -17.48 to -0.03685 |
| B1 vs B14 | 42.21 | 25.23 | Yes | *** | 33.49 to 50.93 |
| B1 vs B15 | 0.3533 | 0.2112 | No | ns | -8.366 to 9.073 |
| B2 vs B3 | -5.707 | 3.41 | No | ns | -14.43 to 3.013 |
| B2 vs B4 | -70.82 | 42.32 | Yes | *** | -79.54 to -62.10 |
| B2 vs B5 | 0.22 | 0.1315 | No | ns | -8.500 to 8.940 |
| B2 vs B6 | 17.18 | 10.26 | Yes | *** | 8.457 to 25.90 |
| B2 vs B7 | 17.64 | 10.54 | Yes | *** | 8.920 to 26.36 |
| B2 vs B8 | 14.75 | 8.817 | Yes | *** | 6.034 to 23.47 |
| B2 vs B9 | 16.43 | 9.819 | Yes | *** | 7.710 to 25.15 |
| B2 vs B10 | 3.683 | 2.201 | No | ns | -5.036 to 12.40 |
| B2 vs B11 | 11.89 | 7.108 | Yes | ** | 3.174 to 20.61 |
| B2 vs B12 | 7.347 | 4.39 | No | ns | -1.373 to 16.07 |
| B2 vs B13 | -37.17 | 22.21 | Yes | *** | -45.89 to -28.45 |
| B2 vs B14 | 13.8 | 8.247 | Yes | *** | 5.080 to 22.52 |
| B2 vs B15 | -28.06 | 16.77 | Yes | *** | -36.78 to -19.34 |
| B3 vs B4 | -65.11 | 38.91 | Yes | *** | -73.83 to -56.39 |
| B3 vs B5 | 5.927 | 3.542 | No | ns | -2.793 to 14.65 |
| B3 vs B6 | 22.88 | 13.68 | Yes | *** | 14.16 to 31.60 |
| B3 vs B7 | 23.35 | 13.95 | Yes | *** | 14.63 to 32.07 |
| B3 vs B8 | 20.46 | 12.23 | Yes | *** | 11.74 to 29.18 |
| B3 vs B9 | 22.14 | 13.23 | Yes | *** | 13.42 to 30.86 |
| B3 vs B10 | 9.39 | 5.612 | Yes | * | 0.6702 to 18.11 |
| B3 vs B11 | 17.6 | 10.52 | Yes | *** | 8.880 to 26.32 |
| B3 vs B12 | 13.05 | 7.801 | Yes | *** | 4.334 to 21.77 |
| B3 vs B13 | -31.46 | 18.8 | Yes | *** | -40.18 to -22.74 |
| B3 vs B14 | 19.51 | 11.66 | Yes | *** | 10.79 to 28.23 |
| B3 vs B15 | -22.35 | 13.36 | Yes | *** | -31.07 to -13.63 |
| B4 vs B5 | 71.04 | 42.45 | Yes | *** | 62.32 to 79.76 |
| B4 vs B6 | 87.99 | 52.59 | Yes | *** | 79.27 to 96.71 |
| B4 vs B7 | 88.46 | 52.86 | Yes | *** | 79.74 to 97.18 |
| B4 vs B8 | 85.57 | 51.14 | Yes | *** | 76.85 to 94.29 |
| B4 vs B9 | 87.25 | 52.14 | Yes | *** | 78.53 to 95.97 |
| B4 vs B10 | 74.5 | 44.52 | Yes | *** | 65.78 to 83.22 |
| B4 vs B11 | 82.71 | 49.43 | Yes | *** | 73.99 to 91.43 |
| B4 vs B12 | 78.16 | 46.71 | Yes | *** | 69.44 to 86.88 |
| B4 vs B13 | 33.65 | 20.11 | Yes | *** | 24.93 to 42.37 |
| B4 vs B14 | 84.62 | 50.57 | Yes | *** | 75.90 to 93.34 |
| B4 vs B15 | 42.76 | 25.55 | Yes | *** | 34.04 to 51.48 |
| B5 vs B6 | 16.96 | 10.13 | Yes | *** | 8.237 to 25.68 |
| B5 vs B7 | 17.42 | 10.41 | Yes | *** | 8.700 to 26.14 |
| B5 vs B8 | 14.53 | 8.685 | Yes | *** | 5.814 to 23.25 |
| B5 vs B9 | 16.21 | 9.687 | Yes | *** | 7.490 to 24.93 |
| B5 vs B10 | 3.463 | 2.07 | No | ns | -5.256 to 12.18 |
| B5 vs B11 | 11.67 | 6.976 | Yes | ** | 2.954 to 20.39 |
| B5 vs B12 | 7.127 | 4.259 | No | ns | -1.593 to 15.85 |
| B5 vs B13 | -37.39 | 22.34 | Yes | *** | -46.11 to -28.67 |
| B5 vs B14 | 13.58 | 8.115 | Yes | *** | 4.860 to 22.30 |
| B5 vs B15 | -28.28 | 16.9 | Yes | *** | -37.00 to -19.56 |
| B6 vs B7 | 0.4633 | 0.2769 | No | ns | -8.256 to 9.183 |
| B6 vs B8 | -2.423 | 1.448 | No | ns | -11.14 to 6.296 |
| B6 vs B9 | -0.7467 | 0.4462 | No | ns | -9.466 to 7.973 |
| B6 vs B10 | -13.49 | 8.064 | Yes | *** | -22.21 to -4.774 |
| B6 vs B11 | -5.283 | 3.157 | No | ns | -14.00 to 3.436 |
| B6 vs B12 | -9.83 | 5.874 | Yes | * | -18.55 to -1.110 |
| B6 vs B13 | -54.35 | 32.48 | Yes | *** | -63.07 to -45.63 |
| B6 vs B14 | -3.377 | 2.018 | No | ns | -12.10 to 5.343 |
| B6 vs B15 | -45.24 | 27.03 | Yes | *** | -53.96 to -36.52 |
| B7 vs B8 | -2.887 | 1.725 | No | ns | -11.61 to 5.833 |
| B7 vs B9 | -1.21 | 0.7231 | No | ns | -9.930 to 7.510 |
| B7 vs B10 | -13.96 | 8.341 | Yes | *** | -22.68 to -5.237 |
| B7 vs B11 | -5.747 | 3.434 | No | ns | -14.47 to 2.973 |
| B7 vs B12 | -10.29 | 6.151 | Yes | ** | -19.01 to -1.574 |
| B7 vs B13 | -54.81 | 32.75 | Yes | *** | -63.53 to -46.09 |
| B7 vs B14 | -3.84 | 2.295 | No | ns | -12.56 to 4.880 |
| B7 vs B15 | -45.7 | 27.31 | Yes | *** | -54.42 to -36.98 |
| B8 vs B9 | 1.677 | 1.002 | No | ns | -7.043 to 10.40 |
| B8 vs B10 | -11.07 | 6.615 | Yes | ** | -19.79 to -2.350 |
| B8 vs B11 | -2.86 | 1.709 | No | ns | -11.58 to 5.860 |
| B8 vs B12 | -7.407 | 4.426 | No | ns | -16.13 to 1.313 |
| B8 vs B13 | -51.92 | 31.03 | Yes | *** | -60.64 to -43.20 |
| B8 vs B14 | -0.9533 | 0.5697 | No | ns | -9.673 to 7.766 |
| B8 vs B15 | -42.81 | 25.59 | Yes | *** | -51.53 to -34.09 |
| B9 vs B10 | -12.75 | 7.617 | Yes | *** | -21.47 to -4.027 |
| B9 vs B11 | -4.537 | 2.711 | No | ns | -13.26 to 4.183 |
| B9 vs B12 | -9.083 | 5.428 | Yes | * | -17.80 to -0.3635 |
| B9 vs B13 | -53.6 | 32.03 | Yes | *** | -62.32 to -44.88 |
| B9 vs B14 | -2.63 | 1.572 | No | ns | -11.35 to 6.090 |
| B9 vs B15 | -44.49 | 26.59 | Yes | *** | -53.21 to -35.77 |
| B10 vs B11 | 8.21 | 4.906 | No | ns | -0.5098 to 16.93 |
| B10 vs B12 | 3.663 | 2.189 | No | ns | -5.056 to 12.38 |
| B10 vs B13 | -40.85 | 24.41 | Yes | *** | -49.57 to -32.13 |
| B10 vs B14 | 10.12 | 6.046 | Yes | * | 1.397 to 18.84 |
| B10 vs B15 | -31.74 | 18.97 | Yes | *** | -40.46 to -23.02 |
| B11 vs B12 | -4.547 | 2.717 | No | ns | -13.27 to 4.173 |
| B11 vs B13 | -49.06 | 29.32 | Yes | *** | -57.78 to -40.34 |
| B11 vs B14 | 1.907 | 1.139 | No | ns | -6.813 to 10.63 |
| B11 vs B15 | -39.95 | 23.88 | Yes | *** | -48.67 to -31.23 |
| B12 vs B13 | -44.52 | 26.6 | Yes | *** | -53.24 to -35.80 |
| B12 vs B14 | 6.453 | 3.857 | No | ns | -2.266 to 15.17 |
| B12 vs B15 | -35.41 | 21.16 | Yes | *** | -44.13 to -26.69 |
| B13 vs B14 | 50.97 | 30.46 | Yes | *** | 42.25 to 59.69 |
| B13 vs B15 | 9.11 | 5.444 | Yes | * | 0.3902 to 17.83 |
| B14 vs B15 | -41.86 | 25.02 | Yes | *** | -50.58 to -33.14 |

TABLE S3: Statistical analysis of disintegration time of different batches conducted by a one-way analysis of variance (One-way ANOVA), with Tukey post hoc test.

| **One-way analysis of variance** | | | | | |
| --- | --- | --- | --- | --- | --- |
| P value | | < 0.0001 | |  |  |
| P value summary | | **** | |  |  |
| Are means signif. Different?  (P < 0.05) | | Yes | |  |  |
| Number of groups | | 15 | |  |  |
| F | | 93.33 | |  |  |
| R square | | 0.9776 | |  |  |
|  | |  | |  |  |
| ANOVA Table | | SS | | df | MS |
| Treatment (between columns) | | 7927 | | 14 | 566.2 |
| Residual (within columns) | | 182 | | 30 | 6.067 |
| Total | | 8109 | | 44 |  |
| **Tukey's Multiple Comparison Test** | |  | |  |  |
| Tests | Mean Diff. | q | Significant?  (P < 0.05?) | Summary | 95% CI of diff |
| B1 vs B2 | 12.33 | 8.673 | Yes | *** | 4.923 to 19.74 |
| B1 vs B3 | 8 | 5.626 | Yes | * | 0.5897 to 15.41 |
| B1 vs B4 | -24.67 | 17.35 | Yes | *** | -32.08 to -17.26 |
| B1 vs B5 | 9.333 | 6.563 | Yes | ** | 1.923 to 16.74 |
| B1 vs B6 | 23.33 | 16.41 | Yes | *** | 15.92 to 30.74 |
| B1 vs B7 | 22 | 15.47 | Yes | *** | 14.59 to 29.41 |
| B1 vs B8 | 20.67 | 14.53 | Yes | *** | 13.26 to 28.08 |
| B1 vs B9 | 24 | 16.88 | Yes | *** | 16.59 to 31.41 |
| B1 vs B10 | 18.33 | 12.89 | Yes | *** | 10.92 to 25.74 |
| B1 vs B11 | 9.667 | 6.798 | Yes | ** | 2.256 to 17.08 |
| B1 vs B12 | 16.67 | 11.72 | Yes | *** | 9.256 to 24.08 |
| B1 vs B13 | -8.667 | 6.094 | Yes | * | -16.08 to -1.256 |
| B1 vs B14 | 22 | 15.47 | Yes | *** | 14.59 to 29.41 |
| B1 vs B15 | 0.3333 | 0.2344 | No | ns | -7.077 to 7.744 |
| B2 vs B3 | -4.333 | 3.047 | No | ns | -11.74 to 3.077 |
| B2 vs B4 | -37 | 26.02 | Yes | *** | -44.41 to -29.59 |
| B2 vs B5 | -3 | 2.11 | No | ns | -10.41 to 4.410 |
| B2 vs B6 | 11 | 7.735 | Yes | *** | 3.590 to 18.41 |
| B2 vs B7 | 9.667 | 6.798 | Yes | ** | 2.256 to 17.08 |
| B2 vs B8 | 8.333 | 5.86 | Yes | * | 0.9230 to 15.74 |
| B2 vs B9 | 11.67 | 8.204 | Yes | *** | 4.256 to 19.08 |
| B2 vs B10 | 6 | 4.219 | No | ns | -1.410 to 13.41 |
| B2 vs B11 | -2.667 | 1.875 | No | ns | -10.08 to 4.744 |
| B2 vs B12 | 4.333 | 3.047 | No | ns | -3.077 to 11.74 |
| B2 vs B13 | -21 | 14.77 | Yes | *** | -28.41 to -13.59 |
| B2 vs B14 | 9.667 | 6.798 | Yes | ** | 2.256 to 17.08 |
| B2 vs B15 | -12 | 8.439 | Yes | *** | -19.41 to -4.590 |
| B3 vs B4 | -32.67 | 22.97 | Yes | *** | -40.08 to -25.26 |
| B3 vs B5 | 1.333 | 0.9376 | No | ns | -6.077 to 8.744 |
| B3 vs B6 | 15.33 | 10.78 | Yes | *** | 7.923 to 22.74 |
| B3 vs B7 | 14 | 9.845 | Yes | *** | 6.590 to 21.41 |
| B3 vs B8 | 12.67 | 8.907 | Yes | *** | 5.256 to 20.08 |
| B3 vs B9 | 16 | 11.25 | Yes | *** | 8.590 to 23.41 |
| B3 vs B10 | 10.33 | 7.267 | Yes | ** | 2.923 to 17.74 |
| B3 vs B11 | 1.667 | 1.172 | No | ns | -5.744 to 9.077 |
| B3 vs B12 | 8.667 | 6.094 | Yes | * | 1.256 to 16.08 |
| B3 vs B13 | -16.67 | 11.72 | Yes | *** | -24.08 to -9.256 |
| B3 vs B14 | 14 | 9.845 | Yes | *** | 6.590 to 21.41 |
| B3 vs B15 | -7.667 | 5.391 | Yes | * | -15.08 to -0.2564 |
| B4 vs B5 | 34 | 23.91 | Yes | *** | 26.59 to 41.41 |
| B4 vs B6 | 48 | 33.75 | Yes | *** | 40.59 to 55.41 |
| B4 vs B7 | 46.67 | 32.82 | Yes | *** | 39.26 to 54.08 |
| B4 vs B8 | 45.33 | 31.88 | Yes | *** | 37.92 to 52.74 |
| B4 vs B9 | 48.67 | 34.22 | Yes | *** | 41.26 to 56.08 |
| B4 vs B10 | 43 | 30.24 | Yes | *** | 35.59 to 50.41 |
| B4 vs B11 | 34.33 | 24.14 | Yes | *** | 26.92 to 41.74 |
| B4 vs B12 | 41.33 | 29.07 | Yes | *** | 33.92 to 48.74 |
| B4 vs B13 | 16 | 11.25 | Yes | *** | 8.590 to 23.41 |
| B4 vs B14 | 46.67 | 32.82 | Yes | *** | 39.26 to 54.08 |
| B4 vs B15 | 25 | 17.58 | Yes | *** | 17.59 to 32.41 |
| B5 vs B6 | 14 | 9.845 | Yes | *** | 6.590 to 21.41 |
| B5 vs B7 | 12.67 | 8.907 | Yes | *** | 5.256 to 20.08 |
| B5 vs B8 | 11.33 | 7.97 | Yes | *** | 3.923 to 18.74 |
| B5 vs B9 | 14.67 | 10.31 | Yes | *** | 7.256 to 22.08 |
| B5 vs B10 | 9 | 6.329 | Yes | ** | 1.590 to 16.41 |
| B5 vs B11 | 0.3333 | 0.2344 | No | ns | -7.077 to 7.744 |
| B5 vs B12 | 7.333 | 5.157 | No | ns | -0.07696 to 14.74 |
| B5 vs B13 | -18 | 12.66 | Yes | *** | -25.41 to -10.59 |
| B5 vs B14 | 12.67 | 8.907 | Yes | *** | 5.256 to 20.08 |
| B5 vs B15 | -9 | 6.329 | Yes | ** | -16.41 to -1.590 |
| B6 vs B7 | -1.333 | 0.9376 | No | ns | -8.744 to 6.077 |
| B6 vs B8 | -2.667 | 1.875 | No | ns | -10.08 to 4.744 |
| B6 vs B9 | 0.6667 | 0.4688 | No | ns | -6.744 to 8.077 |
| B6 vs B10 | -5 | 3.516 | No | ns | -12.41 to 2.410 |
| B6 vs B11 | -13.67 | 9.611 | Yes | *** | -21.08 to -6.256 |
| B6 vs B12 | -6.667 | 4.688 | No | ns | -14.08 to 0.7436 |
| B6 vs B13 | -32 | 22.5 | Yes | *** | -39.41 to -24.59 |
| B6 vs B14 | -1.333 | 0.9376 | No | ns | -8.744 to 6.077 |
| B6 vs B15 | -23 | 16.17 | Yes | *** | -30.41 to -15.59 |
| B7 vs B8 | -1.333 | 0.9376 | No | ns | -8.744 to 6.077 |
| B7 vs B9 | 2 | 1.406 | No | ns | -5.410 to 9.410 |
| B7 vs B10 | -3.667 | 2.578 | No | ns | -11.08 to 3.744 |
| B7 vs B11 | -12.33 | 8.673 | Yes | *** | -19.74 to -4.923 |
| B7 vs B12 | -5.333 | 3.75 | No | ns | -12.74 to 2.077 |
| B7 vs B13 | -30.67 | 21.57 | Yes | *** | -38.08 to -23.26 |
| B7 vs B14 | 0 | 0 | No | ns | -7.410 to 7.410 |
| B7 vs B15 | -21.67 | 15.24 | Yes | *** | -29.08 to -14.26 |
| B8 vs B9 | 3.333 | 2.344 | No | ns | -4.077 to 10.74 |
| B8 vs B10 | -2.333 | 1.641 | No | ns | -9.744 to 5.077 |
| B8 vs B11 | -11 | 7.735 | Yes | *** | -18.41 to -3.590 |
| B8 vs B12 | -4 | 2.813 | No | ns | -11.41 to 3.410 |
| B8 vs B13 | -29.33 | 20.63 | Yes | *** | -36.74 to -21.92 |
| B8 vs B14 | 1.333 | 0.9376 | No | ns | -6.077 to 8.744 |
| B8 vs B15 | -20.33 | 14.3 | Yes | *** | -27.74 to -12.92 |
| B9 vs B10 | -5.667 | 3.985 | No | ns | -13.08 to 1.744 |
| B9 vs B11 | -14.33 | 10.08 | Yes | *** | -21.74 to -6.923 |
| B9 vs B12 | -7.333 | 5.157 | No | ns | -14.74 to 0.07696 |
| B9 vs B13 | -32.67 | 22.97 | Yes | *** | -40.08 to -25.26 |
| B9 vs B14 | -2 | 1.406 | No | ns | -9.410 to 5.410 |
| B9 vs B15 | -23.67 | 16.64 | Yes | *** | -31.08 to -16.26 |
| B10 vs B11 | -8.667 | 6.094 | Yes | * | -16.08 to -1.256 |
| B10 vs B12 | -1.667 | 1.172 | No | ns | -9.077 to 5.744 |
| B10 vs B13 | -27 | 18.99 | Yes | *** | -34.41 to -19.59 |
| B10 vs B14 | 3.667 | 2.578 | No | ns | -3.744 to 11.08 |
| B10 vs B15 | -18 | 12.66 | Yes | *** | -25.41 to -10.59 |
| B11 vs B12 | 7 | 4.922 | No | ns | -0.4103 to 14.41 |
| B11 vs B13 | -18.33 | 12.89 | Yes | *** | -25.74 to -10.92 |
| B11 vs B14 | 12.33 | 8.673 | Yes | *** | 4.923 to 19.74 |
| B11 vs B15 | -9.333 | 6.563 | Yes | ** | -16.74 to -1.923 |
| B12 vs B13 | -25.33 | 17.81 | Yes | *** | -32.74 to -17.92 |
| B12 vs B14 | 5.333 | 3.75 | No | ns | -2.077 to 12.74 |
| B12 vs B15 | -16.33 | 11.49 | Yes | *** | -23.74 to -8.923 |
| B13 vs B14 | 30.67 | 21.57 | Yes | *** | 23.26 to 38.08 |
| B13 vs B15 | 9 | 6.329 | Yes | ** | 1.590 to 16.41 |
| B14 vs B15 | -21.67 | 15.24 | Yes | *** | -29.08 to -14.26 |

TABLE S4: Statistical analysis of water absorption ratio (R) of different batches conducted by a one-way analysis of variance (One-way ANOVA), with Tukey post hoc test.

| **One-way analysis of variance** | | | | | |
| --- | --- | --- | --- | --- | --- |
| P value | | < 0.0001 | |  |  |
| P value summary | | **** | |  |  |
| Are means signif. Different?  (P < 0.05) | | Yes | |  |  |
| Number of groups | | 15 | |  |  |
| F | | 325.8 | |  |  |
| R square | | 0.9935 | |  |  |
|  | |  | |  |  |
| ANOVA Table | | SS | | df | MS |
| Treatment (between columns) | | 8606 | | 14 | 614.7 |
| Residual (within columns) | | 56.61 | | 30 | 1.887 |
| Total | | 8663 | | 44 |  |
| **Tukey's Multiple Comparison Test** | |  | |  |  |
| Tests | Mean Diff. | q | Significant?  (P < 0.05?) | Summary | 95% CI of diff |
| B1 vs B2 | -6.148 | 7.752 | Yes | *** | -10.28 to -2.016 |
| B1 vs B3 | -3.377 | 4.258 | No | ns | -7.510 to 0.7555 |
| B1 vs B4 | 20.48 | 25.82 | Yes | *** | 16.35 to 24.61 |
| B1 vs B5 | -8.631 | 10.88 | Yes | *** | -12.76 to -4.499 |
| B1 vs B6 | -18.61 | 23.46 | Yes | *** | -22.74 to -14.48 |
| B1 vs B7 | -15.17 | 19.13 | Yes | *** | -19.30 to -11.04 |
| B1 vs B8 | -17.03 | 21.48 | Yes | *** | -21.16 to -12.90 |
| B1 vs B9 | -19.07 | 24.05 | Yes | *** | -23.20 to -14.94 |
| B1 vs B10 | -12.06 | 15.2 | Yes | *** | -16.19 to -7.925 |
| B1 vs B11 | -9.638 | 12.15 | Yes | *** | -13.77 to -5.505 |
| B1 vs B12 | -12.38 | 15.61 | Yes | *** | -16.51 to -8.245 |
| B1 vs B13 | 22.01 | 27.76 | Yes | *** | 17.88 to 26.15 |
| B1 vs B14 | -10.73 | 13.53 | Yes | *** | -14.87 to -6.601 |
| B1 vs B15 | 20.68 | 26.08 | Yes | *** | 16.55 to 24.82 |
| B2 vs B3 | 2.771 | 3.494 | No | ns | -1.362 to 6.904 |
| B2 vs B4 | 26.63 | 33.57 | Yes | *** | 22.49 to 30.76 |
| B2 vs B5 | -2.483 | 3.131 | No | ns | -6.616 to 1.649 |
| B2 vs B6 | -12.46 | 15.71 | Yes | *** | -16.59 to -8.327 |
| B2 vs B7 | -9.022 | 11.38 | Yes | *** | -13.15 to -4.889 |
| B2 vs B8 | -10.88 | 13.72 | Yes | *** | -15.02 to -6.750 |
| B2 vs B9 | -12.92 | 16.29 | Yes | *** | -17.06 to -8.790 |
| B2 vs B10 | -5.909 | 7.451 | Yes | *** | -10.04 to -1.777 |
| B2 vs B11 | -3.49 | 4.401 | No | ns | -7.623 to 0.6428 |
| B2 vs B12 | -6.23 | 7.855 | Yes | *** | -10.36 to -2.097 |
| B2 vs B13 | 28.16 | 35.51 | Yes | *** | 24.03 to 32.29 |
| B2 vs B14 | -4.585 | 5.781 | Yes | * | -8.718 to -0.4523 |
| B2 vs B15 | 26.83 | 33.83 | Yes | *** | 22.70 to 30.96 |
| B3 vs B4 | 23.86 | 30.08 | Yes | *** | 19.72 to 27.99 |
| B3 vs B5 | -5.254 | 6.625 | Yes | ** | -9.387 to -1.122 |
| B3 vs B6 | -15.23 | 19.2 | Yes | *** | -19.36 to -11.10 |
| B3 vs B7 | -11.79 | 14.87 | Yes | *** | -15.93 to -7.660 |
| B3 vs B8 | -13.65 | 17.22 | Yes | *** | -17.79 to -9.522 |
| B3 vs B9 | -15.69 | 19.79 | Yes | *** | -19.83 to -11.56 |
| B3 vs B10 | -8.68 | 10.95 | Yes | *** | -12.81 to -4.548 |
| B3 vs B11 | -6.261 | 7.895 | Yes | *** | -10.39 to -2.128 |
| B3 vs B12 | -9.001 | 11.35 | Yes | *** | -13.13 to -4.868 |
| B3 vs B13 | 25.39 | 32.02 | Yes | *** | 21.26 to 29.52 |
| B3 vs B14 | -7.356 | 9.275 | Yes | *** | -11.49 to -3.223 |
| B3 vs B15 | 24.06 | 30.34 | Yes | *** | 19.93 to 28.19 |
| B4 vs B5 | -29.11 | 36.7 | Yes | *** | -33.24 to -24.98 |
| B4 vs B6 | -39.09 | 49.28 | Yes | *** | -43.22 to -34.95 |
| B4 vs B7 | -35.65 | 44.95 | Yes | *** | -39.78 to -31.52 |
| B4 vs B8 | -37.51 | 47.3 | Yes | *** | -41.64 to -33.38 |
| B4 vs B9 | -39.55 | 49.87 | Yes | *** | -43.68 to -35.42 |
| B4 vs B10 | -32.54 | 41.02 | Yes | *** | -36.67 to -28.40 |
| B4 vs B11 | -30.12 | 37.97 | Yes | *** | -34.25 to -25.98 |
| B4 vs B12 | -32.86 | 41.43 | Yes | *** | -36.99 to -28.72 |
| B4 vs B13 | 1.536 | 1.937 | No | ns | -2.597 to 5.669 |
| B4 vs B14 | -31.21 | 39.35 | Yes | *** | -35.34 to -27.08 |
| B4 vs B15 | 0.2056 | 0.2592 | No | ns | -3.927 to 4.338 |
| B5 vs B6 | -9.977 | 12.58 | Yes | *** | -14.11 to -5.844 |
| B5 vs B7 | -6.538 | 8.244 | Yes | *** | -10.67 to -2.406 |
| B5 vs B8 | -8.4 | 10.59 | Yes | *** | -12.53 to -4.267 |
| B5 vs B9 | -10.44 | 13.16 | Yes | *** | -14.57 to -6.307 |
| B5 vs B10 | -3.426 | 4.32 | No | ns | -7.559 to 0.7065 |
| B5 vs B11 | -1.007 | 1.269 | No | ns | -5.139 to 3.126 |
| B5 vs B12 | -3.747 | 4.724 | No | ns | -7.879 to 0.3861 |
| B5 vs B13 | 30.65 | 38.64 | Yes | *** | 26.51 to 34.78 |
| B5 vs B14 | -2.102 | 2.65 | No | ns | -6.234 to 2.031 |
| B5 vs B15 | 29.32 | 36.96 | Yes | *** | 25.18 to 33.45 |
| B6 vs B7 | 3.438 | 4.335 | No | ns | -0.6946 to 7.571 |
| B6 vs B8 | 1.577 | 1.988 | No | ns | -2.556 to 5.709 |
| B6 vs B9 | -0.4629 | 0.5837 | No | ns | -4.596 to 3.670 |
| B6 vs B10 | 6.55 | 8.26 | Yes | *** | 2.418 to 10.68 |
| B6 vs B11 | 8.97 | 11.31 | Yes | *** | 4.837 to 13.10 |
| B6 vs B12 | 6.23 | 7.856 | Yes | *** | 2.097 to 10.36 |
| B6 vs B13 | 40.62 | 51.22 | Yes | *** | 36.49 to 44.75 |
| B6 vs B14 | 7.875 | 9.929 | Yes | *** | 3.742 to 12.01 |
| B6 vs B15 | 39.29 | 49.54 | Yes | *** | 35.16 to 43.42 |
| B7 vs B8 | -1.861 | 2.347 | No | ns | -5.994 to 2.271 |
| B7 vs B9 | -3.901 | 4.919 | No | ns | -8.034 to 0.2317 |
| B7 vs B10 | 3.112 | 3.924 | No | ns | -1.020 to 7.245 |
| B7 vs B11 | 5.532 | 6.975 | Yes | ** | 1.399 to 9.664 |
| B7 vs B12 | 2.792 | 3.52 | No | ns | -1.341 to 6.925 |
| B7 vs B13 | 37.18 | 46.89 | Yes | *** | 33.05 to 41.32 |
| B7 vs B14 | 4.437 | 5.594 | Yes | * | 0.3040 to 8.569 |
| B7 vs B15 | 35.85 | 45.21 | Yes | *** | 31.72 to 39.99 |
| B8 vs B9 | -2.04 | 2.572 | No | ns | -6.172 to 2.093 |
| B8 vs B10 | 4.974 | 6.271 | Yes | ** | 0.8410 to 9.106 |
| B8 vs B11 | 7.393 | 9.322 | Yes | *** | 3.260 to 11.53 |
| B8 vs B12 | 4.653 | 5.867 | Yes | * | 0.5206 to 8.786 |
| B8 vs B13 | 39.05 | 49.23 | Yes | *** | 34.91 to 43.18 |
| B8 vs B14 | 6.298 | 7.941 | Yes | *** | 2.165 to 10.43 |
| B8 vs B15 | 37.71 | 47.56 | Yes | *** | 33.58 to 41.85 |
| B9 vs B10 | 7.013 | 8.843 | Yes | *** | 2.881 to 11.15 |
| B9 vs B11 | 9.433 | 11.89 | Yes | *** | 5.300 to 13.57 |
| B9 vs B12 | 6.693 | 8.439 | Yes | *** | 2.560 to 10.83 |
| B9 vs B13 | 41.08 | 51.8 | Yes | *** | 36.95 to 45.22 |
| B9 vs B14 | 8.338 | 10.51 | Yes | *** | 4.205 to 12.47 |
| B9 vs B15 | 39.75 | 50.13 | Yes | *** | 35.62 to 43.89 |
| B10 vs B11 | 2.419 | 3.051 | No | ns | -1.713 to 6.552 |
| B10 vs B12 | -0.3204 | 0.404 | No | ns | -4.453 to 3.812 |
| B10 vs B13 | 34.07 | 42.96 | Yes | *** | 29.94 to 38.20 |
| B10 vs B14 | 1.324 | 1.67 | No | ns | -2.808 to 5.457 |
| B10 vs B15 | 32.74 | 41.28 | Yes | *** | 28.61 to 36.87 |
| B11 vs B12 | -2.74 | 3.455 | No | ns | -6.873 to 1.393 |
| B11 vs B13 | 31.65 | 39.91 | Yes | *** | 27.52 to 35.78 |
| B11 vs B14 | -1.095 | 1.381 | No | ns | -5.228 to 3.038 |
| B11 vs B15 | 30.32 | 38.23 | Yes | *** | 26.19 to 34.45 |
| B12 vs B13 | 34.39 | 43.37 | Yes | *** | 30.26 to 38.52 |
| B12 vs B14 | 1.645 | 2.074 | No | ns | -2.488 to 5.778 |
| B12 vs B15 | 33.06 | 41.69 | Yes | *** | 28.93 to 37.19 |
| B13 vs B14 | -32.75 | 41.29 | Yes | *** | -36.88 to -28.61 |
| B13 vs B15 | -1.33 | 1.678 | No | ns | -5.463 to 2.802 |
| B14 vs B15 | 31.42 | 39.61 | Yes | *** | 27.28 to 35.55 |
